# Supplementary material for: Learning curve and surgical outcome of robotic assisted colorectal surgery with ERAS program
Source: Sci Rep. 2022 Nov 29;12:20566. doi: 10.1038/s41598-022-24665-w (PMC9709162; doi:10.1038/s41598-022-24665-w)
Supplement: Supplementary file 1 — Supplementary Information 1. [file 41598_2022_24665_MOESM1_ESM.zip › LSC Raw data-Ñ[▒K/073/H_ñw╝╨░O▒Kñσ.pdf]

正式摘要(電子簽章：000000188055979)<br>W52 / W52 ☐ 英文標題(注意:無英文姓名-請補病人基本檔!)	☐【讀取】憑證	

臺中榮民總醫院

出院病歷摘要

轉入號 : 轉出號 :

[redacted]

(8)流水編號	
(9)入院日期	109/03/18 (2020) 直腸外科 W52 - 172
(10)轉科日期	
(11)出院日期	109/03/25 (2020) 住院天數計 7 日

診斷

- 入院 : 1.Sigmoid colon cancer
  2.Right ureteral stone
- 出院 : 1.Adenocarcinoma of sigmoid colon, s/p laparoscopic anterior resection on 2020/3/19, pT3N2a
  2.Right ureteral stone, s/p laser URSL+ bil. D-J insertion on 2020/3/19

主訴

- intermittent diarrhea more than 10 times/ day for at least 1 month

病史

- This 53 years old male has history of GERD and gastritis.
  He complained of intermittent diarrhea more than 10 times/ day for at least 1 month. He ever went to LMD, but symptom did not improve. So he wen to 大甲光田H. in 2020/2 where upper GI scope revealed gastritis, LGI scope revealed S-colon cancer at 20cm FAV, with pathology proved adenocarcinoma. CT showed sigmoid colon wall thickening. Then he came to our OPD for second opinion. This time, he was admitted for laproscopic AR.

體檢發現

- Anal inspection:
  Recto digital examination:
  Sigmoidoscopy:
  Anoscopy:
  Abdomen:
  - General appearance :
    - easy looking , conscious clear , without cardiopulmonary distress
    - Vital signs自動帶入date 體溫(BT) 脈搏(P) 呼吸(R) 血壓(BP) 身高(BH) 體重(BW) BMI
      20200318 36.7℃ 97次/分 17次/分 113/91mmHg 170cm 79kg 27.3
    - Vital signs: B.P. 113/91 mmHg , P.R. 97 /min , R.R. 17 /min , B.T. 36.7 ℃
  - HEENT :
    - Eyes: no anemic conjunctiva, no icteric sclera, normal EOM
    - ENT: no otorrhea, no rhinorrhea, no erythematous mucosa, no injected tonsil, no deformity
  - Neck: supple, freely movable, trachea midline,no deformity, no engorged jugular vein , no palpable thyroid or lymph node enlargement
  - Chest:
    - Inspection: Grossly normal, symmetric and fully expansion, no accessory muscle used, no operation scar, no spider angiomata, no gynecomastia
    - Palpation: no palpable mass or nodule, no subcutaneous emphysema, no tenderness
    - Ausculation: clear breathing sound
  - Heart:
    - Palpation: no thrill, no heave
    - Percussion: normal heart size
    - Ausculation: regular heart beat, normal S1, S2, no gallop, no murmur

- Extremities: No deformity, full ranged of motion without limitation, no clubbing finger or nail cyanosis, no pitting edema, no ulcer or pigmentation, no obvious skin lesion, no varicose vein, no bone, joint or muscle tenderness

CRS CONDITION

- Karnofsky performance status: (100) Normal, no complaints, no evidence of disease
- PE of Abdomen:
- CEA: 1.34 ng/ml
- Imaging Studies:
- Colonoscope report: S-colon cancer
- Figures:
  1. colon & rectum

手術日期及方法

- 手術日期及方法20200319 Laparoscopic Anterior resection and anastomosis - malignant
  20200319 1.Ureter, Ureteroscopy & removal of stone-with Nd-YAG laser and double J stent,right

  2.Ureter, Double J insertion, left

住院治療經過

- After admission, preop survey was done which was within acceptable range. Laparoscopic AR+ureterolithotripsy+bil. D-J insertion was done on 3/19. He tolerated the operation well. On POD1, we removed NG and then try water, and he tried liquid diet since POD2 without discomfort. After he could eat well, and stool passage was smooth, we removed penrose drain and arranged MBD and OPD f/u for him.

合併症

一般檢查

- 累積報告 :

DATE	TIME	ALB	TP	BIL.T	BIL.D	ALKP	AST	ALT	LDH	CHOL	TG	HDL-C	LDL	UA	NH3	AMY
1090319	1737	4.4		0.7	0.2	74	25	28								
1090323	0924	3.4		0.5	0.1		20									

DATE	TIME	WBC	RBC	HGB	HCT	MCV	PLT	BLST	NEUT	BAND	SEG	LYM	MON	EOS	BAS
1090319	1736	7000	5.14	15.9	47.1	91.6	146		86.5			12.4	1.0	0.0	0.1
1090323	0901	4600		14.3					51.6			29.8	7.8	10.4	0.4

DATE	TIME	NA	K	CL	CA	BUN	CREAT	Mg	P	CRP	ESR	PCT	LIPASE	LACTATE
1090319	1737	140	4.5	104	9.3	11	1.04							
1090323	0924	142	3.9			7	0.90							

DATE	TIME	GLUCOSE	PAB	TRF	ZN	CU	KETONE	CK	CKMB	PT-P	PT-C	APTT-P	APTT-C	TRO-T
1090319	1735	132												

- 放射線報告 :檢驗項目 **:ABDOMEN (KUB),SUPINE** 報告日期 **:1090323**

  主　訴：
  Result:
  Finding:
  > Nonspecific bowel gas pattern
  > The right renal contour is partially obscured by bowel gas
  > No significant bony destructions
  > Surgical clips and autosuture retention in left abdominal and pelvic region
  > Radiopaque densities over right renal region, renal stones cannot be ruled out. Suggest f/u.
  > S/P ostomy over left lower abdomen
  > On bilateral double-J catheters
  > On drainage tube over pelvic region
  Impression:
  As above description.

  檢驗項目 **:CHEST PA VIEW** 報告日期 **:1090310**

  主　訴：
  Result:
  Finding:
  > Heart size within normal limits
  > Mild increased interstitial infiltration over bilateral lungs
  > Degenerative change of T-L spine
  Impression:
  As above description.

特殊檢查

病理報告

- 活體組織切片病理報告　手術日期：**109/03/19**　病理號：**S0908312**

診斷醫師:吳致瑩 報告日期:109/03/27
Pathologic diagnosis:
1. Rectum and sigmoid colon, lapa anterior resection --- Adenocarcinoma, moderately differentiated. Tumor invaded to pericolorectal soft tissue and metastasized to mesocolic lymph nodes (4/27). The bilateral cut ends are free of tumor.
2. IMA root, dissection --- Unremarkable fibrovascular tissue and lymph node (0/1).
3. Colon, cut end, distal, resection --- Chronic inflammation.
4. "Lymph node", IMA, dissection --- Unremarkable fibroadipose tissue. No lymph node seen.
Ancillary study for diagnosis:
IHC stains for MLH1, MSH2, PMS2 and MSH6 are done on section D. All above markers are preserved.
Prognostic and predictive factor:
1. Tumor size: 4.2x3.5 cm.
2. Depth of invasion: Tumor invades to pericolorectal tissue.
3. Histologic grade: G2: Moderately differentiated.
4. Angiolymphatic invasion: Not identified.
5. Perineural invasion: Not identified.
6. Tumor deposits (discontinuous extramural extension): Not identified.
7. Macroscopic tumor perforation: Not identified.
8. Surgical Margins: a) Proximal Margin: Uninvolved by invasive carcinoma, distance of tumor from margin: 5.7 cm. b) Distal Margin: Uninvolved by invasive carcinoma, distance of tumor from margin: 3.4 cm. c) Radial or Mesenteric Margin: Uninvolved by invasive carcinoma, distance of tumor from margin: 3 cm.
9. Lymph node status: Involved (4/28).
10. Extranodal involvement: Not identified.
11. Tumor regression grading S/P CCRT: No known presurgical therapy.
12. Type of polyp in which invasive carcinoma arose: Not identified.
13. Additional pathologic findings: Not identified.
14. Pathological TNM Stage: pT3N2a(According to the eighth edition, American Joint Committee on Cancer Staging Guidelines for Tumors).
15. TNM descriptors: Not applicable.
Gross description:
The specimen consists of 1) a segment of colon, 13 cm in length and 3.5 cm in proximal cut end circumference, 3.5 cm in distal circumference, and mesocolic soft tissue. A gray tan firm annular elevated and centrally ulcerative tumor, 4.2x3.5 cm, is noted, 3.4 cm to the nearest cut end. On cross section, the tumor invades to the mesocolic soft tissue with 30 mm to the radial margin. The both cut ends are free of tumor grossly. 2) IMA root, a piece of tan yellow soft tissue, 3.7x2.8x1 cm. 3) distal cut end, a piece of tan mucosa-covered soft tissue, 1.8x1.5x0.8 cm. 4) IMA LN, a piece, 1x0.7x0.3 cm. Representative part for section: A) proximal cut end B) distal cut end C-E) tumor F) colon G) radial margin H-K) specimen 2-4 L-P) lymph nodes.
Note:
1. The interpretation of MMR panel and recommended studies are as below: (1) No loss of nuclear expression of MMR proteins: low probability of microsatellite instability-high (MSI-H) . (2) Please note: There are exceptions to the above IHC interpretations. These results should not be considered in isolation, and clinical correlation with genetic counseling is recommended to assess the need for germline testing.
2. This case has been peer reviewed by two doctors.
#T-59300_2 #M-81403_2 2 1105 S0000F
#T-C4560_2 #M-81406_2 2 1105 000000

其它

出院時情況

- 3.改門診治療

出院指示

-

藥名	劑量	劑量單位	用法	頻次	數量	數量單位	現況	天數	單位
Ultracet tab	1	TAB	PO	Q6HPRN	20	TAB	TH	5	天

出院計劃

主治醫師 2062F 蔣鋒帆 / 住院醫師 2303K 陳映菁

正式摘要(電子簽章：000000188055979)

W52 / W52

doctorRank:B
